# Differences in genetics and microenvironment of lung adenocarcinoma patients with or without *TP53* mutation

**DOI:** 10.1186/s12890-021-01671-8

**Published:** 2021-10-11

**Authors:** Dejun Zeng, Zhengyang Hu, Yanjun Yi, Besskaya Valeria, Guangyao Shan, Zhencong Chen, Cheng Zhan, Miao Lin, Zongwu Lin, Qun Wang

**Affiliations:** Department of Thoracic Surgery, Zhongshan Hospital, Fudan University, 180 Fenglin Road, Shanghai, 200032 China

**Keywords:** *TP53*, Lung adenocarcinoma, Mutation, Genome, Microenvironment

## Abstract

**Background:**

Differences in genetics and microenvironment of LUAD patients with or without *TP53* mutation were analyzed to illustrate the role of *TP53* mutation within the carcinogenesis of LUAD, which will provide new concepts for the treatment of LUAD.

**Methods:**

In this study, we used genetics and clinical info from the TCGA database, including somatic mutations data, RNA-seq, miRNA-seq, and clinical data. More than one bioinformatics tools were used to analyze the unique genomic pattern of *TP53*-related LUAD.

**Results:**

According to *TP53* gene mutation status, we divided the LUAD patients into two groups, including 265 in the mutant group (MU) and 295 in the wild-type group (WT). 787 significant somatic mutations were detected between the groups, including mutations in *titin (TTN), type 2 ryanodine receptor (RYR2)* and *CUB and Sushi multiple domains 3(CSMD3)*, which were up-regulated in the MU. However, no significant survival difference was observed. At the RNA level, we obtained 923 significantly differentially expressed genes; in the MU, *α-defensin 5(DEFA5), pregnancy-specific glycoprotein 5(PSG5)* and *neuropeptide Y(NPY)* were the most up-regulated genes, *glucose-6-phosphatase (G6PC), alpha-fetoprotein (AFP)* and *carry gametocidal (GC)* were the most down-regulated genes. GSVA analysis revealed 30 significant pathways. Compared with the WT, the expression of 12 pathways in the mutant group was up-regulated, most of which pointed to cell division. There were significant differences in tumor immune infiltrating cells, such as Macrophages M1, T cells CD4 memory activated, Mast cells resting, and Dendritic cells resting. In terms of immune genes, a total of 35 immune-related genes were screened, of which *VGF (VGF nerve growth factor inducible)* and *PGC (peroxisome proliferator-activated receptor gamma coactivator)* were the most significant up-regulated and down-regulated genes, respectively. Research on the expression pattern of immunomodulators found that 9 immune checkpoint molecules and 6 immune costimulatory molecules were considerably wholly different between the two groups.

**Conclusions:**

Taking the mutant group as a reference, LUAD patients in the mutant group had significant differences in somatic mutations, mRNA-seq, miRNA-seq, immune infiltration, and immunomodulators, indicating that *TP53* mutation plays a crucial role in the occurrence and development of LUAD.

**Supplementary Information:**

The online version contains supplementary material available at 10.1186/s12890-021-01671-8.

## Background

According to the study in 2020, among all cancers, the mortality of lung cancer ranks first, with about 1.8 million death occurring, and the incidence of lung cancer ranks second, with more than 2.2 million new cases being diagnosed [1]. Lung adenocarcinoma (LUAD) is the most frequent subtype [2], accounting for more than 40% of all lung cancers [3]. Despite considerable progress in both diagnosis and treatment, the five-year survival of patients with lung cancer remains very poor [4].

*TP53*, coding the supermolecule p53, is located on human chromosome 17p 13.1 and plays a vital role in controlling cell cycle progression, aging, DNA repair and senescence, cell death, and cell metabolism [5–7]. This function is achieved through its wild-type form. Once the *TP53* gene is mutated, it loses its position as a tumor suppressor gene and promotes tumorigenesis at the same time [5, 8]. *TP53* mutation is considered to be the most common kind of gene-specific changes in human cancers and occurs in almost every type of human tumours [9–11]. Unlike other tumor suppressors that are usually inactivated by frameshift or nonsense mutation, most of the *TP53* mutation are missense mutation, and the same is true in lung cancer [9, 12]. Previous studies have shown that *TP53* mutations can promote the metastasis of cancer cells [13], some important somatic mutations can affect the effectiveness of LUAD immunotherapy [14, 15]. In addition, abnormal *TP53* is considered to be an important prognostic factor for no-small-cell carcinoma (NSCLC) [16]. However, to date, it is still unknown how *TP53* mutation affects LUAD patients.

To study the effect of TP53 mutation on LUAD patients, we consistently analyzed changes in somatic mutation data, clinical data, immune infiltration data, and gene expression obtained from the TCGA database. This research will enhance our understanding of *TP53* mutation in LUAD and offer a reference for future studies.

## Materials and methods

### Acquisition of LUAD expression data set

VarScan 2-based somatic mutation data were obtained from the TCGA website (https://portal.gdc.cancer.gov/) (TCGA_LUAD), RNA and miRNA sequencing data, and corresponding clinical data were additionally extracted from this website. After matching with somatic mutation data, the RNA data of 523 patients, miRNA data of 509 patients, and clinical data of 509 patients were finally screened for future research between the groups with or without *TP53* mutation of LUAD patients.

### Somatic mutation analysis

According to *TP53* gene mutation status, the somatic mutation data was divided into a wild-type group (WT, n = 295) and a mutant group (MU, n = 265). The “maftools” package in R (version 4.0.4) was applied to estimate the mutation rate of every gene [17]. The statistical significance threshold was set to an adjusted *p*-value of 0.05.

### Summary statistics of clinical features

All clinical data were analyzed using SPSS statistical analysis software (version 23.0). In the group comparison of categorical variables, Pearson’s chi-square was used, with *p*-value = 0.05 as the cutoff [18].

### Screening for differentially expressed genes (DEGs)

The original data from the TCGA database were screened by removing the data whose average expression value was less than 1 in all samples, the remaining data were normalized by a weighted trimmed average based on a logarithmic ratio method. To obtain DEGs between the groups, the mRNA expression data, miRNA expression data, and IncRNA expression data were analyzed using the R package “edgeR” [19]. Taking the |log2 fold change| > 1.0 and an adjusted *p*-value < 0.05 as the critical value to identify differentially expressed genes. To further investigate the relationship between DEGs, the protein-protein interactions (PPI) network, transcription factor regulatory network, and ceRNA network were constructed and analyzed [20]. Cytoscape software (version 3.7.1) was used to visualized all networks.

To identify the potential biological function differences between WT and MU, R package “clusterProfiler” was used to perform gene ontology (GO) analysis and Kyoto Encyclopedia of Genes and Genomes (KEGG) pathway analysis on the differentially expressed mRNAs [21]. Gene Set Enrichment Analysis (GSEA) was performed via GSEA (version 4.0.3), and Gene Set Variation Analysis (GSVA) was performed via the GSVA software package in the R [22].

### Estimation of tumor immune infiltrating cells

To systematically quantify the proportions of immune cells in the TCGA_LUAD samples, we uploaded a modified TCGA RNA-seq dataset with standard annotation to the CIBERSOPT portal, and ran the LM22 signature matrix at 1000 permutations, which allows for high sensitivity and specific discrimination of *22* human immune phenotypes [23, 24]. Each sample was screened by the R package “Genefilter,” and the statistical significance threshold was set to a *p*-value of 0.05. Then the final CIBERSORT output was analyzed.

### Analysis of immune-related genes

In order to obtained immune-related genes differentially expressed between the groups, we took the intersection of the differentially expressed mRNAs and the immune genes set, downloaded from The ImmPort Shared Data (https://www.immport.org/home). In addition, we compared the expression differences of fifteen immune checkpoint molecules and twenty costimulatory molecules between the groups [25]. The threshold for significance was set as *p*-value < 0.05 and|log2 fold change|> 1.0.

## Results

### Differences in somatic mutations

**Fig. 1:**
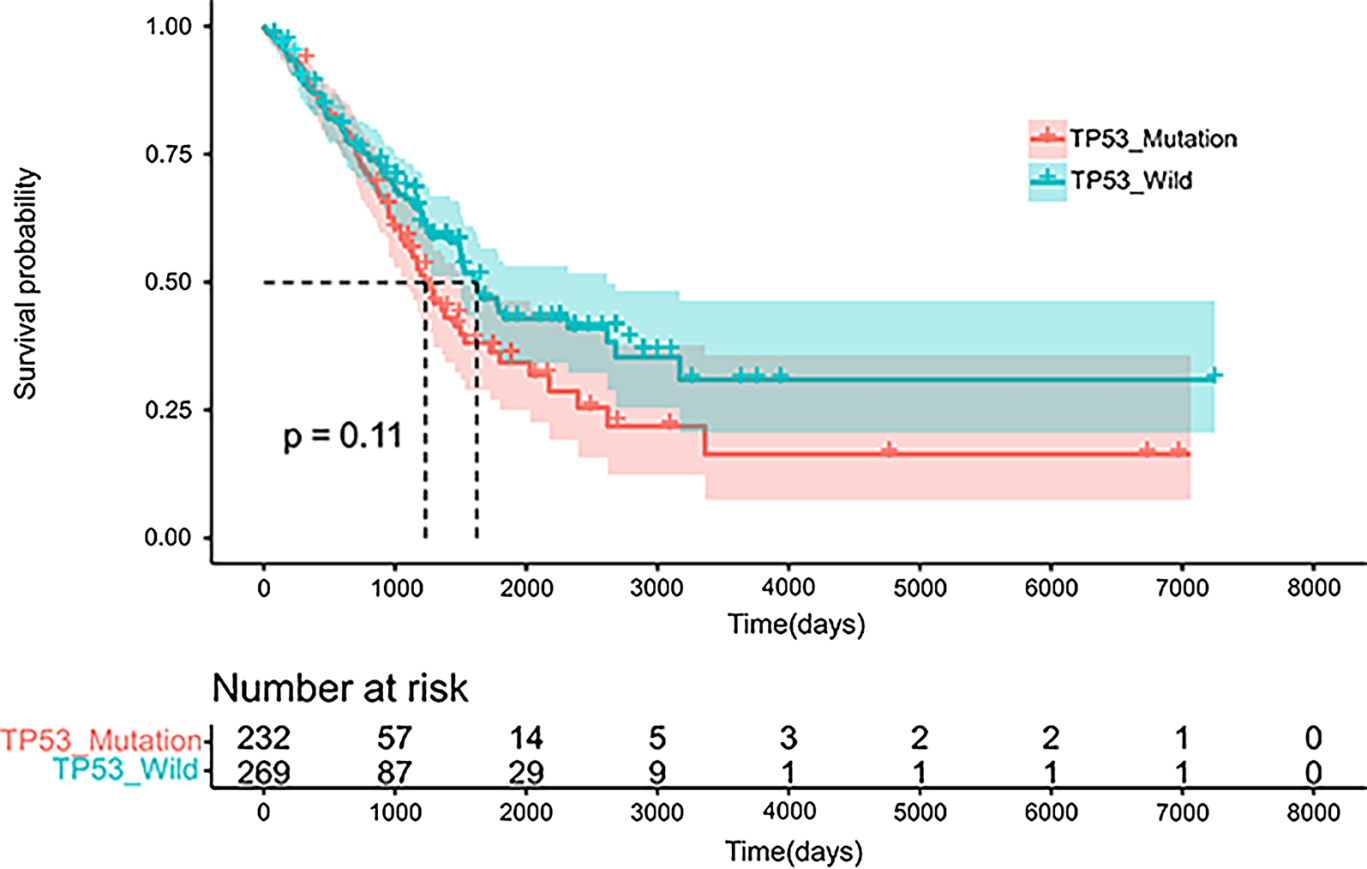
Survival analysis: survival time analysis of 501 patients with *TP53* status

On the basis of the mutation of the TP53 gene in the sample tissues, we divided the somatic mutations data into a wild group (WT, n = 295) and a mutant group (MU, n = 265). We first analyzed the distribution of the somatic mutations between the MU and WT (Additional file 1: Figure S1). To understand the difference in clinical features between the WT (n = 274) and the MU (n = 235), we compared the age, gender, stage, and TNM stage of the two groups (Table 1). The results showed that people younger than or equal to 65 years old accounted for a higher proportion in the MU. However, no significant differences were found in other aspects, such as gender, stage and TNM stage. And no statistically significant difference in survival between the groups was observed (Fig. 1). We also analyzed the proportions of various *TP53* mutation in the MU (Additional file 1: Figure S2A). The highest proportion of mutation was missense mutation, accounting for more than half (61.9%), followed by nonsense mutation (19.71%) and frame_shift_del (9.32%), while the remaining mutation types accounted for less than10%. We also found that the mutation of *TP53* are mainly concentrated in the three domains of the P53 protein, including the Pro-rich domain (PRD), the central DNA binding domain (DBD), and the tetramerization domain (TD), especially the three sites (R158L/P/AFs*12, G245V/S/C, R273L/G/H/C) in the DBD (Additional file 1: Figure S2B).

**Table 1:** Clinical features of the TCGA samples

Characteristics	Mutation (N = 235)		Wild (N = 274)		*p* value
	N	%	N	%	
Age					0.005
≤ 65	127	54.04	109	39.78	
> 65	102	43.41	153	55.84	
NA	6	2.55	12	4.38	
Sex					0.994
Male	109	46.38	127	46.35	
Female	126	53.62	147	53.65	
Stage					0.630
Stage I	120	51.06	155	56.57	
Stage II	60	25.53	59	21.54	
Stage III	38	16.17	45	16.42	
Stage IV	14	5.96	11	4.01	
NA	3	1.28	4	1.46	
T					0.565
T1	75	31.91	93	33.94	
T2	131	55.74	145	52.92	
T3	19	8.09	25	9.12	
T4	8	3.40	11	4.01	
NA	2	0.85	0	0.00	
M					0.190
M0	150	63.83	195	71.17	
M1	14	5.96	11	4.01	
NA	71	30.21	68	24.82	
N					0.384
N0	146	62.13	180	65.69	
N1	50	21.28	47	17.15	
N2	33	14.04	40	14.60	
N3	2	0.85	0	0.00	
NA	4	1.70	7	2.55

**Fig. 2:**
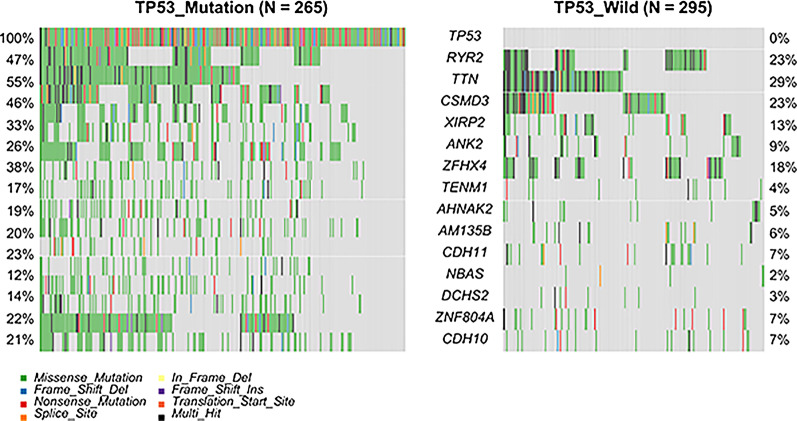
Somatic mutation waterfall map grouped by *TP53* status, the left group corresponded to the *TP53* mutation group, and the right was the *TP53* wild group

Taking FDR < 0.05 as the screening criteria, 787 significant mutations were distinguished between the MU and the WT (Fig. 2). The somatic mutation rates of *titin (TTN*; WT, 29% vs. MU, 55%; *p* < 0.001); *type 2 ryanodine receptor (RYR2*; WT,23% vs. MU,47%; *p* < 0.001); *CUB and Sushi multiple domains 3*(*CSMD3*; WT,23% vs. MU,46%; *p* < 0.001) and *Xin-actin binding repeat containing 2*(*XIRP2*; WT, 13% vs. MU, 33%; *p* < 0.001) were higher in the MU, while *ataxia-telangiectasia mutated (ATM*; WT, 11.2% vs. MU, 3.8%; *p* = 0.011) and *Kirsten rat sarcoma viral oncogene (KRAS*; WT, 30.2% vs. MU, 18.5%; *p* = 0.012) were higher in the WT. Additionally, the mutation rate of the *epidermal growth factor receptor(EGFR)* was not significantly different between the groups. The somatic cell interactions function was performed to detect the correlation between the top 25 genes with different somatic mutation rates (Additional file 1: Figure S3). Except for the closely related *TTN*, most of the mutated genes are mutually exclusive, including the *KRAS*. The results strongly indicated that these mutations might be involved in the occurrence and development of LUAD.

### Differential genes expression (DEGs)

**Fig. 3:**
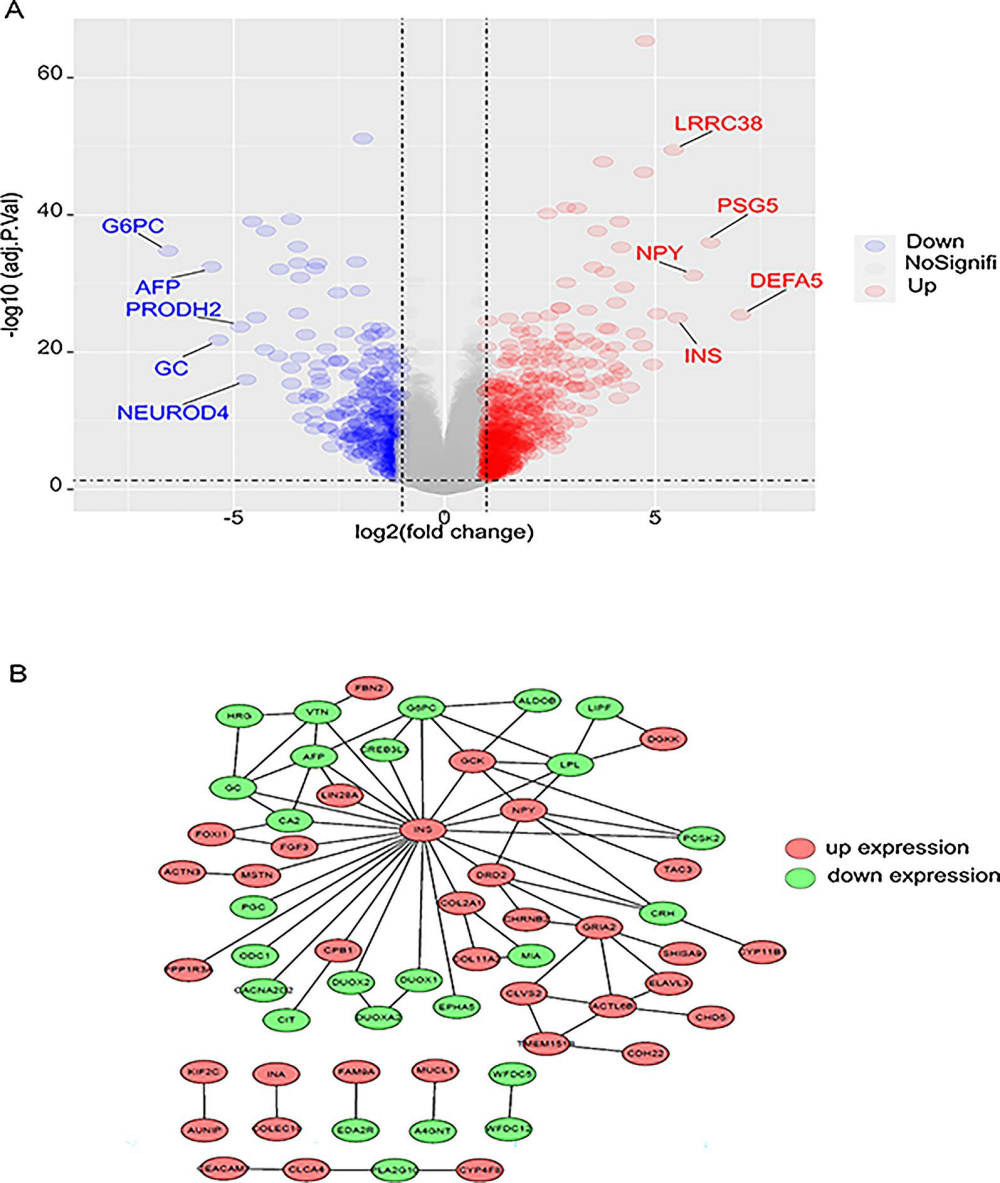
**A** Volcano map of differential gene expression from TCGA database between the groups with or without *TP53* mutation. (Red represents high expression in the group with *TP53* mutation, blue represents high expression in the group without *TP53* mutation). **B** Protein-protein interaction (PPI) network of differently expressed genes

In order to study the impact of *TP53* mutation on gene expression, we matched and grouped the obtained RNA-seq data with somatic mutation data, and then analyzed the differential genes expression between the MU (n = 231) and WT (n = 278). Using the WT as a standard, 542 up-regulated mRNAs and 381 down-regulated mRNAs were detected from the MU. among which *alpha-defensin 5 (DEFA5)* was the most significantly differentially expressed gene (logFC = 7.03, *p* < < 0.001) (Fig. 3A).

To further study the relationship between DEGs, we established and analyzed the protein-protein interactions (PPIs), which was based on the top 100 DEGs. The results showed that *INS, NPY*(logFC = 5.90, *p* < < 0.001), and *AFP* were located in the center of the PPI map (Fig. 3B). We also constructed the transcription factor regulation network of all DEGs and found that the transcription factors *CHX10, S8* and *LHX3* were the hub in the network. (Additional file 1: Figure S4). These genes might be associated with the mutation of TP53 between the groups.

### The correlation between miRNAs and lncRNAs with the mutation of *TP53*

Both miRNAs and lncRNAs play vital roles in epigenetic regulation. The differentially expressed miRNAs and IncRNAs were calculated according to the above method of analyzing mRNAs. 57 up-regulated miRNAs and 17 down-regulated miRNAs were obtained (Additional file 1: Figure S5A). MiR-371a-5p was found to be the most significant up-regulated (logFC = 7.62, *p* < 0.0001), while miR-122-5p was the most down-regulated (logFC = −  3.87, *p* < 0.0001) miRNAs. At the same time, 298 up-regulated and 200 down-regulated lncRNAs were discovered between the two groups (Additional file 1: Figure S5B). Of these IncRNAs, LINC02106 was the most substantially up-regulated (logFC = 4.70, *p* < 0.0001), and AC112495.1 was the most significantly down-regulated (logFC= −  4.94, *p* < 0.0001). Next, a ceRNA network composed of differential mRNAs, miRNAs, and lncRNAs was established. We found that *DLX6-AS1* regulated most miRNAs and mRNAs (Fig. S6), which may be related to the mutation of the *TP53*.

### Gene functional analysis

**Fig. 4:**
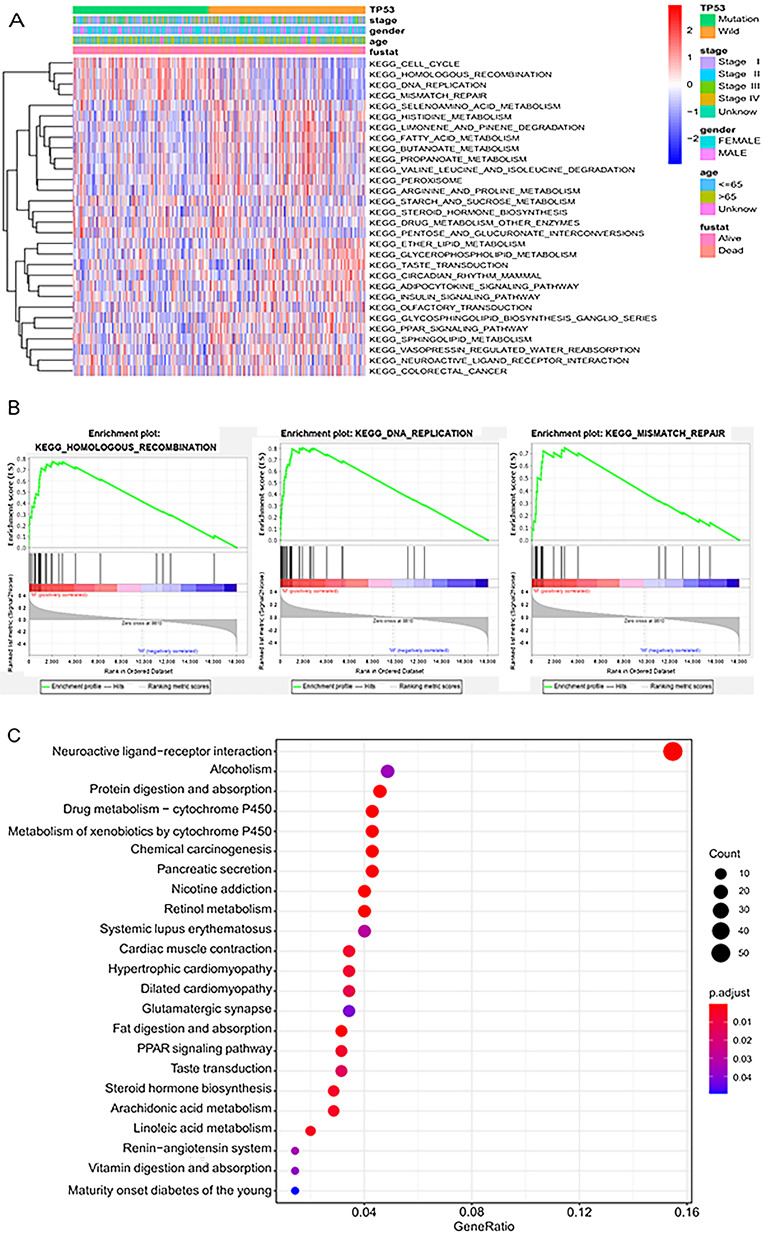
**A** Heatmap of gene set variation analysis for microarray and RNA Seq data (GSVA). **B** The three most significant path ways of Gene Set Enrichment Analysis (GSEA). **C** Barplot of significantly different pathways from KEGG analysis of all aberrant genes

Gene set variation analysis (GSVA) is an unsupervised method of gene set enrichment, which is used to evaluate pathway activity variation in a simple population in an unsupervised manner. GSVA analysis discovered 30 different gene sets between the MU and the WT (*p* < 0.05). Taking the WT as a reference, the expression of 12 pathways was up-regulated in the MU, most of which were associated with cell division, such as cell cycle, homologous recombination, and DNA replication (Fig. 4A). To understand the functions of DEGs, we performed Kyoto Encyclopedia of Genes and Genomes (KEGG) analyses enrichment analysis based on GSEA analysis. Finally, it was found that 12 pathways were enriched in the MU, most of which were associated with DNA, including homologous recombination, DNA replication, and mismatch repair. This indicates that the mutation of the *TP53* gene does have a significant effect on cell division (Fig. 4B).

To characterize 923 differentially expressed mRNAs, Kyoto Encyclopedia of Genes and Genomes (*KEGG*) pathway analysis was performed. The results showed that 23 pathways were enriched, and the significant pathways were closely related to metabolism (Fig. 4C).

Immune cell infiltration landscapes in the WT and MU.

**Fig. 5:**
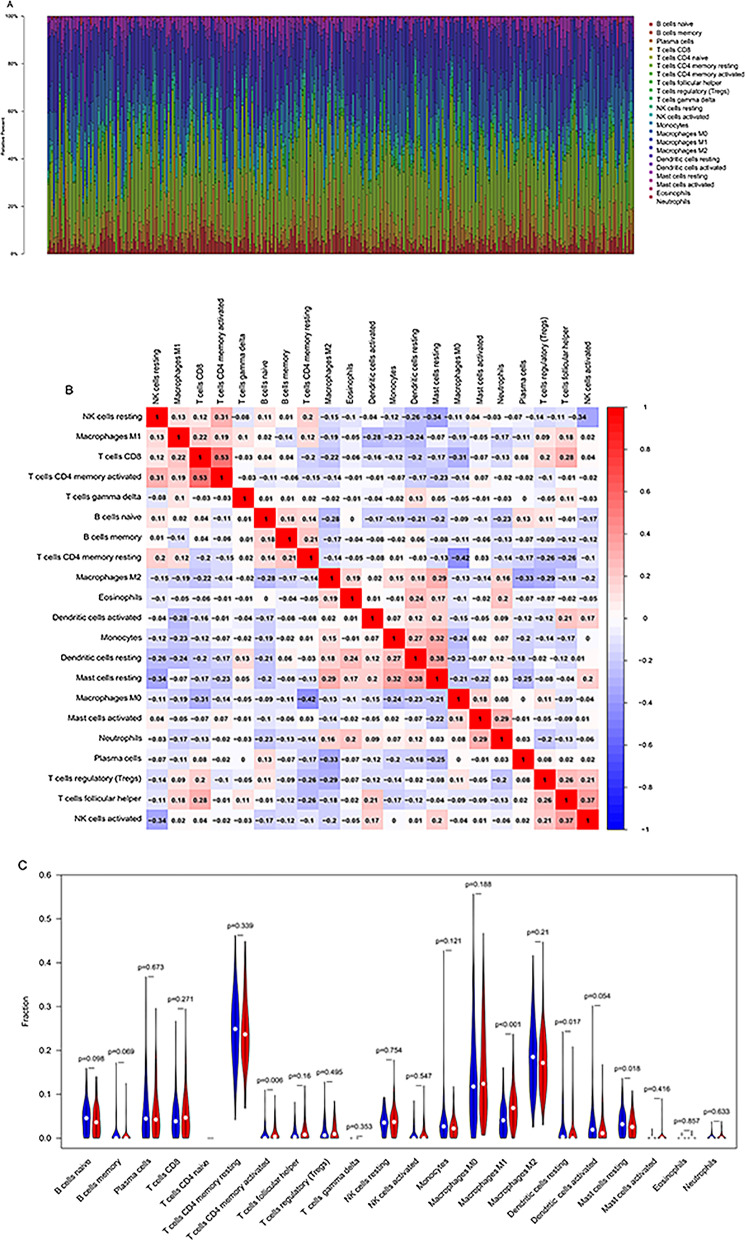
Immune cell infiltration landscapes in patients of LUAD with or without *TP53* mutation. **A** Relative proportions of immune cell infiltration in the wild group and mutant group. **B** Correlation heat map of immune cells. **C** Differences in immune cell infiltration abundances between wild and mutant group

We next investigated the distinction in immune infiltration between the MU and WT. As shown in Fig. 5A, there was a significant difference in the proportion of 22 tumor immune cell types between WT and MU. Additionally, the proportion of immune cells was weak to moderately correlated (Fig. 5B). We also found that Macrophages M1 (*p* < 0.001), T cells CD4 memory activated (*p* = 0.006), Mast cells resting (*p* = 0.018), and Dendritic cells resting (*p* = 0.017) showed significant differences in expression (Fig. 5C). The distinction of immune cell infiltration between the MU and WT might offer new ideas and targets for immunotherapy, which may have a vital clinical significance.

Differences in immune genes.

**Fig. 6:**
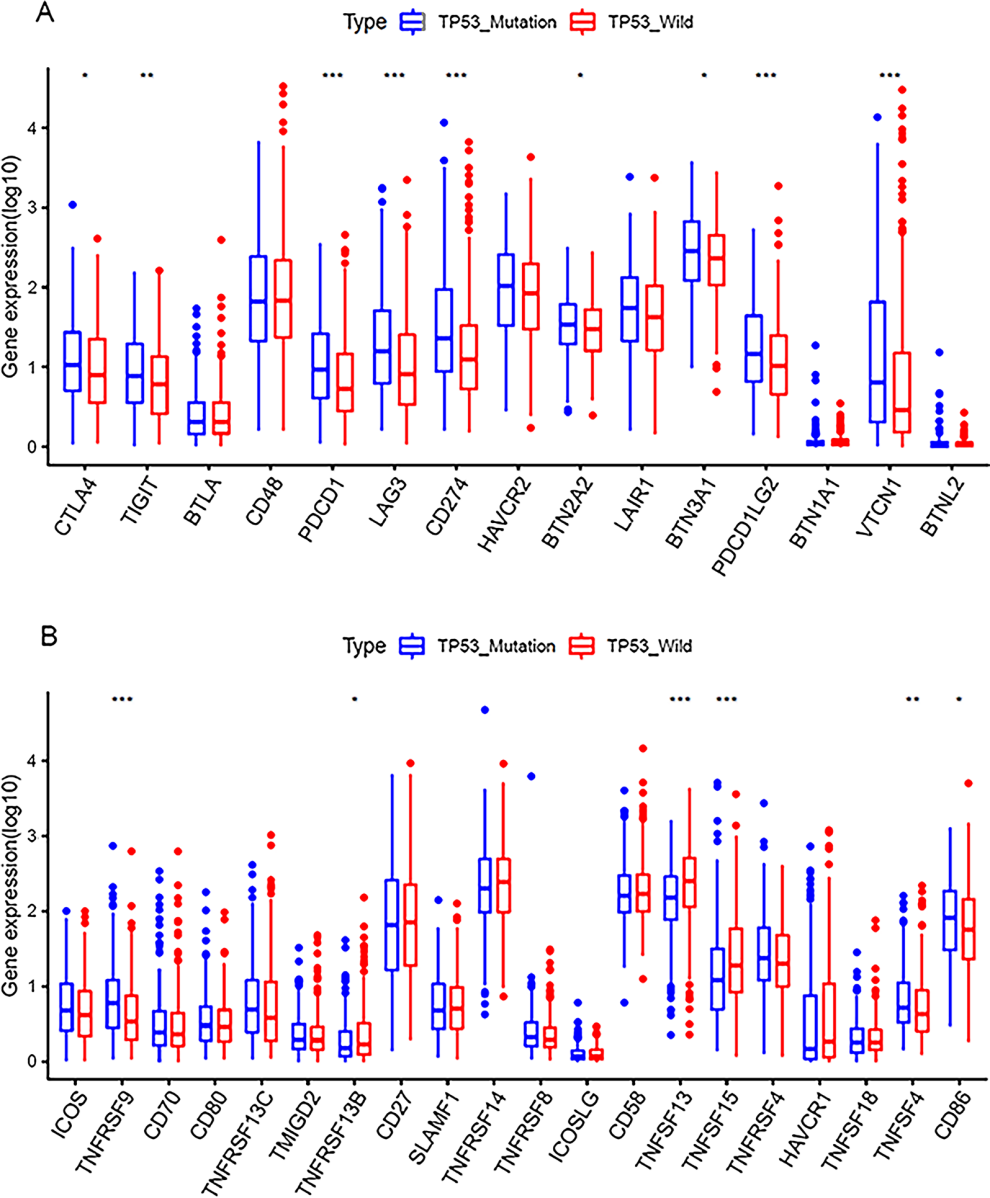
Expression of immune modulators (*represents *P* < 0.05, ** represents *P* < 0.01, ***represents *P* < 0.001). Relative expression level of immune co-inhibitors (**A**) and co-stimulators (**B**)

To describe the impact of TP53 gene mutation on immune-related genes, we first downloaded a list of immune-related genes from The ImmPort Shared Data, took the intersection with the previously obtained differentially expressed mRNAs, and performed the differential analysis of the obtained results, finally screened out 6 up- regulated and 29 down-regulated immune-related genes. (Additional file 1: Figure S7). *VGF* was the most substantially up-regulated (logFC = 1.86, *p* < 0.0001), and *PGC* was the most significantly down-regulated (logFC = - 4.19, *p* < 0.0001).In addition, we investigated the expression patterns of several immunomodulators between the groups, including fifteen immune checkpoint molecules (Fig. 6A) and twenty costimulatory molecules (Fig. 6B). Some highly expressed costimulating and co-inhibitory molecules in MU were observed, such as *PD-1, PD-L1, TNFSF13, and TNFRSF9*. This suggests that these patients may benefit from immunotherapy.

## Discussion

Despite a recent decline in incidence, lung cancer remains the leading cause of death by cancer [26]. Previous studies had shown that compared with the corresponding wild-type tumors, *TP53* mutant NSCLC expressed higher levels of PD-L1 protein, promoted T cell infiltration, and enhanced tumor immunogenicity [27, 28]. However, the characteristics of patients with *TP53* mutation in lung adenocarcinoma are still unclear. In this study, we have first clarified the effect of *TP53* mutation on the microenvironment and genetics of patients with LUAD, which will help us understand the underlying molecular mechanisms and be used clinically in the future.

The entire *TTN* gene consists of 364 exons, located on chromosome 2q31, and its mutation is thought to be related to a variety of skeletal muscle and cardiomyopathy [29]. In our study, we found that the mutation rate of *TTN* was higher in the MU. The previous research has proved that *TTN* and *TP53* mutations may have a combined effect in LUSC, and mutation in the TTN gene show good predictive value in LUSC, but this effect does not apply to LUAD [30]. Another study indicated that the frequency of TTN mutation showed the highest correlation with the response rate to immune checkpoint blockades for individual tumor types, including LUAD [31].

We also studied the associations of mutation with clinical features and outcomes. We found that the *TP53* gene had a higher mutation rate in people younger than or equal to 65 years old, reaching 53.8%, and it was only 40% in people over 65 years.

old, which was consistent with the results of previous studies [32, 33]. We also confirmed that *TP53* mutation did not affect the prognosis of patients with LUAD, it was consistent with the result of the study by Szymanowska, A., et al. [34–39]. However, other studies suggested that patients with mutation of the TP53 gene had a poor prognosis [15, 32, 33, 40, 41]. This phenomenon was worth studying. In fact, only the OS of patients who received specific treatment was considered to be related to the mutation of *TP53*. In contrast, for patients who had not received treatment, the mutation of *TP53* did not affect the prognosis. In addition, we found that there was no difference in *TP53* mutation between genders; both were 47%, the same was true for Marrogi, A. J., et al. [42]

At the same time, we found that the mutation had a significant impact on gene expression, such as *DEFA5* and NPY, which had higher expression in the MU. *DEFA5* is an alpha-defensins [43], produced and secreted by Paneth cells [44]. It was reported that *DEFA5* peptide was highly presented in cancers, including lung cancer [45]. In gastric cancer, the overexpression of *DEFA5* can inhibit cell proliferation and tumor growth [46]. Similarly, in esophageal squamous cell carcinoma, *DEFA5* can inhibit the growth of cancer cells by down-regulating the expression of E-cadherin [47]. All these indicated that *DEFA5* may have a specific tumor inhibitory effect. But further researches are needed to clarify the specific mechanisms of *DEFA5* affecting LUAD. *NPY* gene was not only highly expressed in the MU, but also in a relatively central position in the PPI map. NPY encoded by the *NPY* gene is a 36 amino acid neuropeptide, which is involved in the regulation of a large number of physiological and pathophysiological processes in the cardiopulmonary system, immune system, nervous system and endocrine system [48]. Some studies have shown that high expression of NPY can affect the cell cycle and promote tumor invasion and metastasis [49, 50], also in LUAD [33].

We also studied the changes in the function of differentially expressed genes. The results showed that the effect of mutation on gene function was closely related to cell division. For instance, after GSVA analysis, we found that the cell cycle and homologous recombination were significantly up-regulated in the MU. Regulation of cell cycle is a complicated biological process, and numerous regulatory proteins, including *TP53*, participate in it [51]. Homologous recombination repairs DNA double-strand breaks in S-phase post -replication or G2 in a generally error-free manner [52]. A previous study showed that wild-type *TP53* could inhibit replication-associated homologous recombination [53].

Significantly upregulated and downregulated miRNAs were also identified, such as miR-371a-5p (logFC = 7.62, *p* < < 0.001) and miR-122-5p (logFC = −  3.87, *p* < < 0.001). Previous studies have revealed that miR-371a-5p can affects the MAPK signaling pathway, which is closely related to cell apoptosis and lipid metabolism [54, 55]. In contrast, the overexpression of miR-371a-5p can promote the proliferation and metastasis of cancer cells [56]. Research by Yue, L. and J. Guo et al. showed that miR-371a-5p promoted the development of pancreatic cancer [57]. However, the role of miR-371a-5p in LUAD needs to be further investigated. The low expression of miR-122-5p is more common in the MU in our research. As a tumor suppressor gene [58], it plays a crucial role in inhibiting the metastasis and epithelial-mesenchymal transition of NSCLC [59]. *DLX6-AS1* regulated the most differentially expressed genes. The high expression of DLX6-AS1 is related to the disease stage, positive lymph node metastasis, and poor tumor differentiation in advanced NSCLC [60]. The low expression of *DLX6-AS1* can significantly inhibit the proliferation, migration, and invasion of NSCLC cells and induce apoptosis [61–63]. But in our study, *DLX6-AS1* is highly expressed in the MU.

We have also focused on the relationship between *TP53* mutation and immunity. In terms of immune genes, we found that VGF and PGC are the most apparent up-regulated and down-regulated immune genes. A study indicated that *VGF* significantly promotes the resistance of human lung cancer cells to *EGFR* kinase inhibitors and is also related to the poor survival of patients with LUAD [64]. Matsumoto, T., et al. believed that *VGF* is only expressed in neuroendocrine carcinoma-derived cells and can be used as a new serological diagnostic marker for pulmonary neuroendocrine tumors [65]. *PGC-1α* is a crucial transcription regulator of genes that control energy metabolism and mitochondrial biogenesis through its partner transcription factors: nuclear respiratory factors and PPARs [66]. Overexpression of PGC-1α enhanced the efficacy of PD-1 blockers in lung cancer [67]. Both costimulating and co-inhibitory molecules have higher expression in the MU, including PD1 and PDL1. PD1 is located on lymphocytes, and PDL1 is located on antigen presenting cells. Their interaction leads to tolerance of the immune system to tumor cells. Sun, H., et al. believed that mutant TP53 may enhance PD-L1 expression by activating the newly acquired function of BCL2L1 /JAK3/STAT1 signaling [68]. However, in several reports of anti-PD-1/PD-L1 therapy for NSCLC, the expression of PD-L1 in tumors has been considered to be a standard and predictive biomarker for poor prognosis [14, 69]. Previous studies had shown that the survival outcome of patients with various types of cancer treated by immunotherapy was significantly related to the immune cells infiltrated in the tumor [70]. In our study, we found that macrophages M1 and T cells CD4 memory activated were comparatively upregulated in the MU, while mast cells resting and dendritic cells resting were downregulated. A report indicated that mast cells could promote growth and metastasis by producing IL-1β during LUAD progression [71].

Our study also has some limitations. First, the information from the TCGA database lacks some essential clinicopathological information, such as the patient’s treatment; secondly, to verify our results, another independent cohort study and more in vitro or in vivo studies should be conducted.

## Conclusions

To sum up, our study described the impact of *TP53* gene mutations on the genome and microenvironment in patients with LUAD. Compared with the WT, patients in the MU with LUAD had different microenvironmental RNAs and miRNAs, including immune cell infiltration and immunomodulators. We hope that this study can deepen our understanding of the pathogenesis of *TP53* mutant LUAD and provide a reference for further research.

## Supplementary Information

**Additional file 1**. Supplementary information.

## Data Availability

The data set analyzed in this study can be queried in the TCGA database ( https://portal.gdc.cancer.gov/).

## Abbreviations

LUAD: Lung adenocarcinoma
MU: Mutant group
WT: Wild-type group
TTN: Titin
RYR2: Type 2 ryanodine receptor
CSMD3: CUB and Sushi multiple domains 3
DEFA5: α-defensin 5
PSG5: Pregnancy-specific glycoprotein 5
NPY: Neuropeptide Y
G6PC: Glucose-6-phosphatase
AFP: Alpha-fetoprotein
GC: Carry gametocidal
VGF: VGF nerve growth factor inducible
PGC: Peroxisome proliferator-activated receptor gamma coactivator
NSCLC: Non-small cell carcinoma
DGEs: Differentially expressed genes
PPI: Protein–protein interactions
GO: Gene ontology
KEGG: Kyoto Encyclopedia of Genes and Genomes
GSEA: Gene Set Enrichment Analysis
GSVA: Gene Set Variation Analysis
PRD: Pro-rich domain
DBD: The central DNA binding domain
TD: Tetramerization domain
XIRP2: Xin-actin binding repeat containing 2
ATM: Ataxia-telangiectasia mutated
KRAS: Kirsten rat sarcoma viral oncogene
EGFR: Epidermal growth factor receptor
