# Supplementary material for: Differences in genetics and microenvironment of lung adenocarcinoma patients with or without TP53 mutation
Source: BMC Pulm Med. 2021 Oct 11;21:316. doi: 10.1186/s12890-021-01671-8 (PMC8507221; doi:10.1186/s12890-021-01671-8)
Supplement: Supplementary file 1 — Additional file 1. Supplementary information. [file 12890_2021_1671_MOESM1_ESM.doc]

**Supplementary**


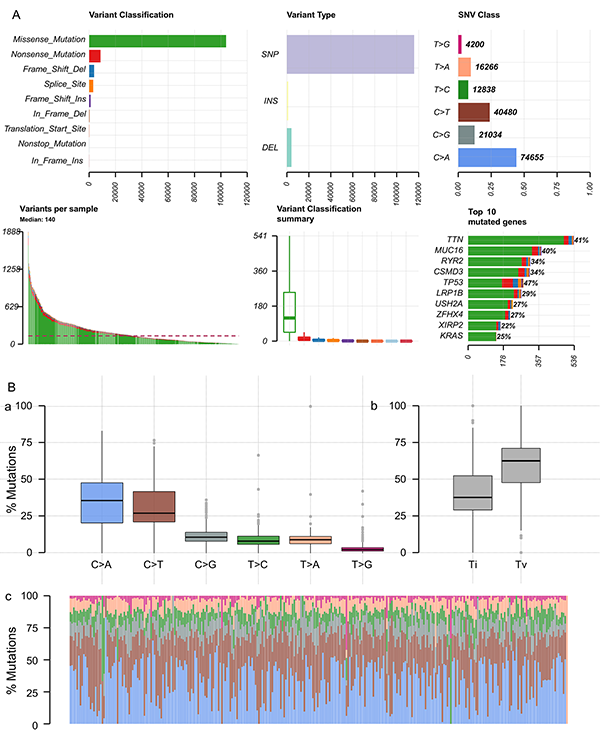


**Figure S1:** The summary of the LUAD patients’ somatic mutation data, (A) displayed number of variants in each sample as a stacked bar plot and variant types as a boxplot summarized; (B) classified SNPs into transitions and transversions, (a) showed the overall distribution of the six different transformations, (b) classified the SNPs as transitions (Ti) and transversions (Tv), showing their proportion, (c) stacked bar graph of the percent conversion in each sample.


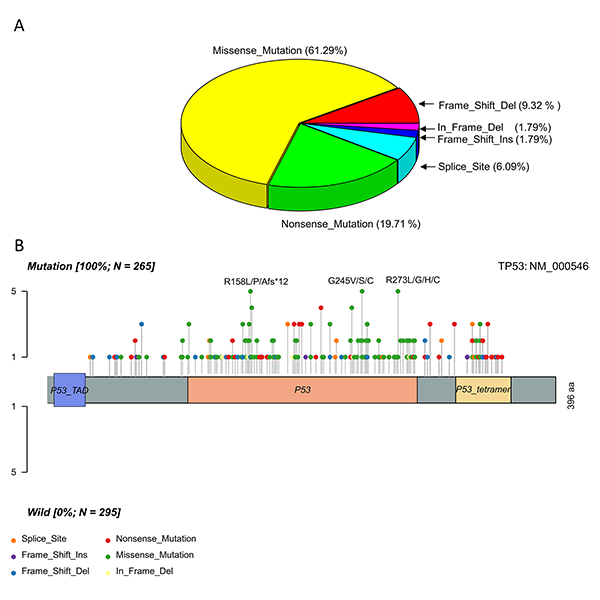


**Figure S2:**(A) Proportion of various *TP53* mutations in MU, (B) Map of P53 protein structure and mutation sites.

**
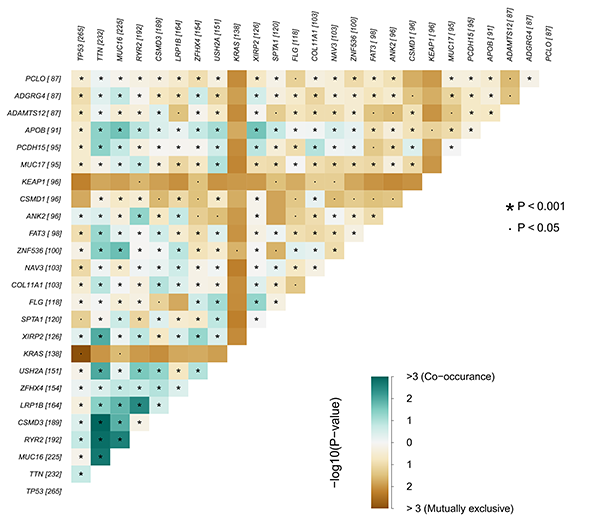
**

**Figure S3:** Exclusive/co-occurrence event analysis on top 25 differently mutated genes.

**
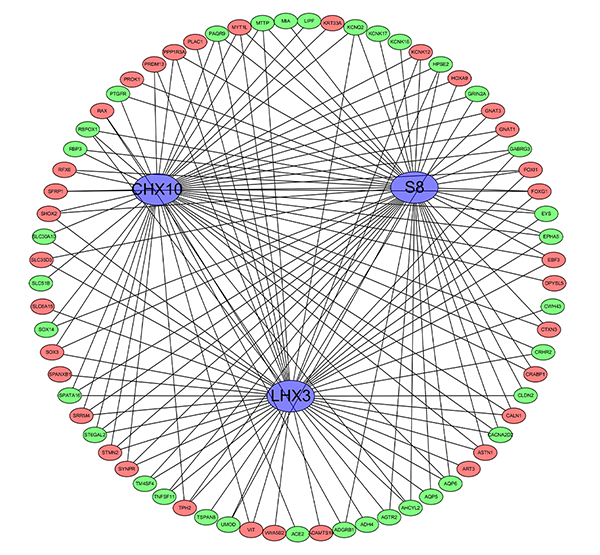
Figure S4:** Transcription factor regulatory network of aberrantly expressed genes in the TCGA database, green dots represent regulatory factors, red dots represent up-regulated mRNA, blue dots represent down-regulated mRNA.


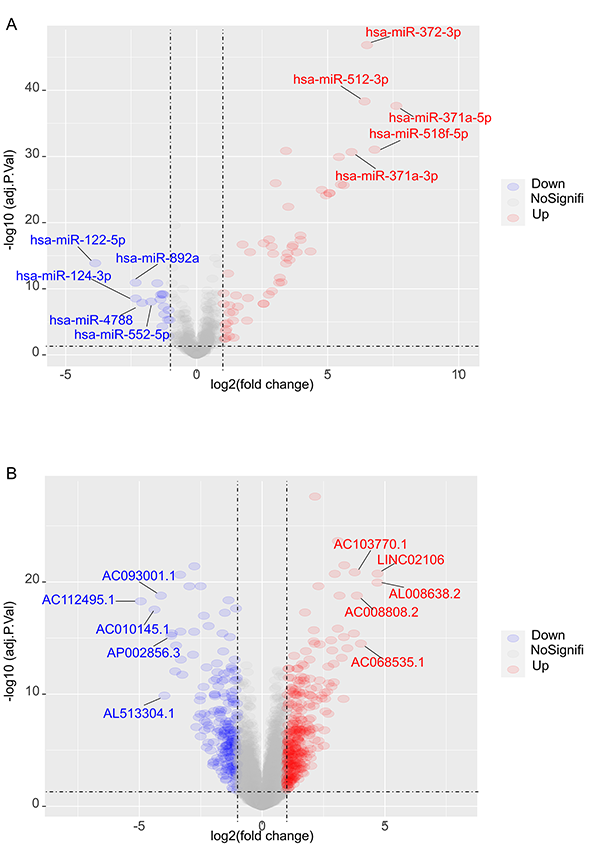


**Figure S5:** Volcano map of differential gene expression from TCGA database between the groups with or without *TP53* mutation. (Red represents high expression in the group with *TP53* mutation, blue represents high expression in the group without *TP53* mutation) (A) differential miRNAs expression (B) differential lncRNAs expression.


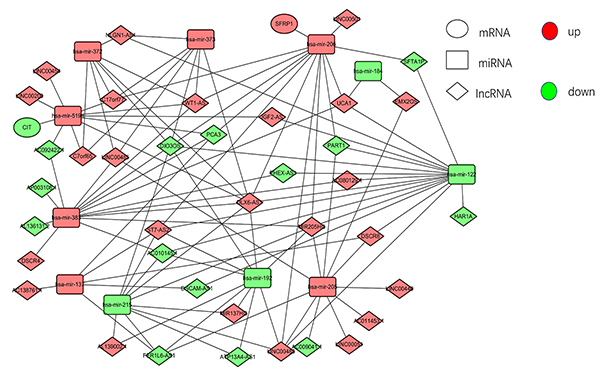


**Figure S6:** CeRNA network of diﬀerential lncRNA-diﬀerential miRNA-diﬀerentially expressed gene pairs.


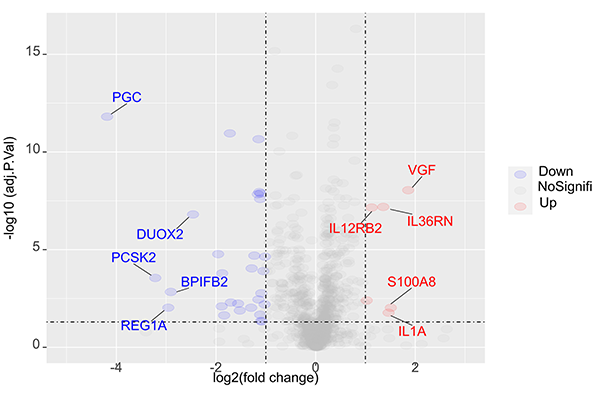


**Figure S7:** Volcano map of differential immune gene expression from TCGA database between the groups with or without *TP53* mutation. (Red represents high expression in the group with *TP53* mutation, blue represents high expression in the group without *TP53* mutation).
